# Supplementary material for: Strongly coupled interface ferroelectricity and interface superconductivity in amorphous LaAlO3/KTaO3(111)
Source: Nat Commun. 2026 Feb 14;17:2805. doi: 10.1038/s41467-026-69641-4 (PMC13022375; doi:10.1038/s41467-026-69641-4)
Supplement: Supplementary file 1 — Supplementary Information [file 41467_2026_69641_MOESM1_ESM.pdf]

Strongly coupled interface ferroelectricity and interface superconductivity in a-$LaAlO_3$/$KTaO_3$(111)

M.D. Dong[1, 2, 3†], X.B. Cheng[1, 2, 3†], M. Zhang[4†], J. Wu[1, 2, 3*]

[1]*Department of Physics, School of Science, Westlake University, Hangzhou 310024, China*

[2]*Research Center for Industries of the Future, Westlake University, Hangzhou310024, China*

[3]*Key Laboratory for Quantum Materials of Zhejiang Province, School of Science, Westlake University, Hangzhou, 310024, China*

[4]*School of Physics, Zhejiang University, Hangzhou 310027, China*

[†]*These authors contributed equally to this work.*

**Author to whom correspondence should be addressed: wujie@westlake.edu.cn*

Supplementary Information

1. Supplementary characterizations using scanning transmission electron microscopy (STEM)

To characterize the cation interdiffusion across amorphous $LaAlO_3$/$KTaO_3$ (LAO/KTO) interface, we carried out the energy dispersive X-ray spectroscopy measurement using scanning transmission electron microscopy (EDS-STEM) (Fig. S1). K and Ta atoms are clearly resolved, indicating that KTO is a single crystal with aligned atoms. In contrast, La and Al atoms do not form a regular lattice, indicating that LAO is amorphous. The distribution of atoms verifies that the interfacial caution interdiffusion is within 1 nm.

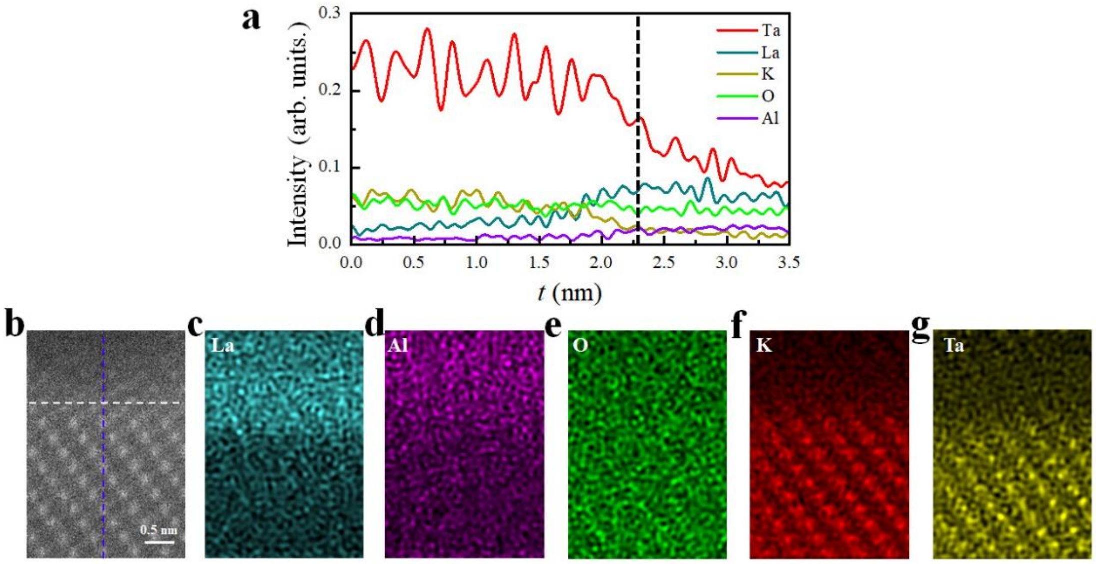

Figure S1 │ EDS-STEM mappings. **a,** The line profile (corresponding to the purple dashed line in panel **b**) of different elements across the LAO/KTO interface. The location of the interface is indicated by the dashed line. **b**, The HAADF-STEM image. **c-g**, EDS mappings for the La, Al, O, Ta, and K elements, respectively. The interfacial cation interdiffusion is within 1 nm.

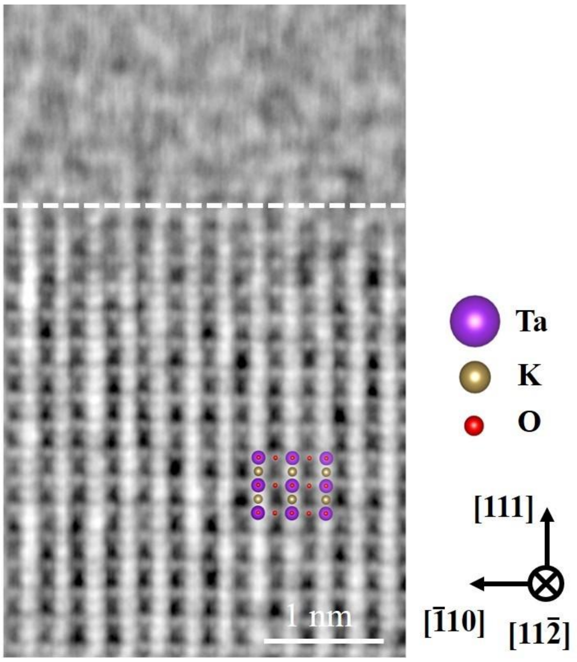

Figure S2 │ The iDPC-STEM image of LAO/KTO(111) with electrons incident along the KTO $[11\bar{2}]$ direction. The interface is denoted by the dashed line. Atomic displacement and oxygen vacancies, which are clearly visible for electrons incident along the KTO $[\bar{1}10]$ direction (Fig. 1), are not distinguishable from this viewing angle. This evidences that the K-Ta displacement is entirely along KTO [110] direction and thus has no projection in the $(11\bar{2})$ plane. Concomitantly, oxygen vacancies residing at the K-O plane (Fig. 1), overlap with K atoms in position and hence is invisible from this viewing angle. Meanwhile, there is no sign of oxygen vacancies in the Ta-O plane at the interface.

A clear K displacement along [110] is visible in the (-110) plane (Fig. 1). We rotated the viewing angle to check for components orthogonal to [110], examining the (11-2) plane, which is perpendicular to the (-110) plane. No displacement was observed along [-110] (Fig. S2), confirming that the displacement lies entirely along [110].

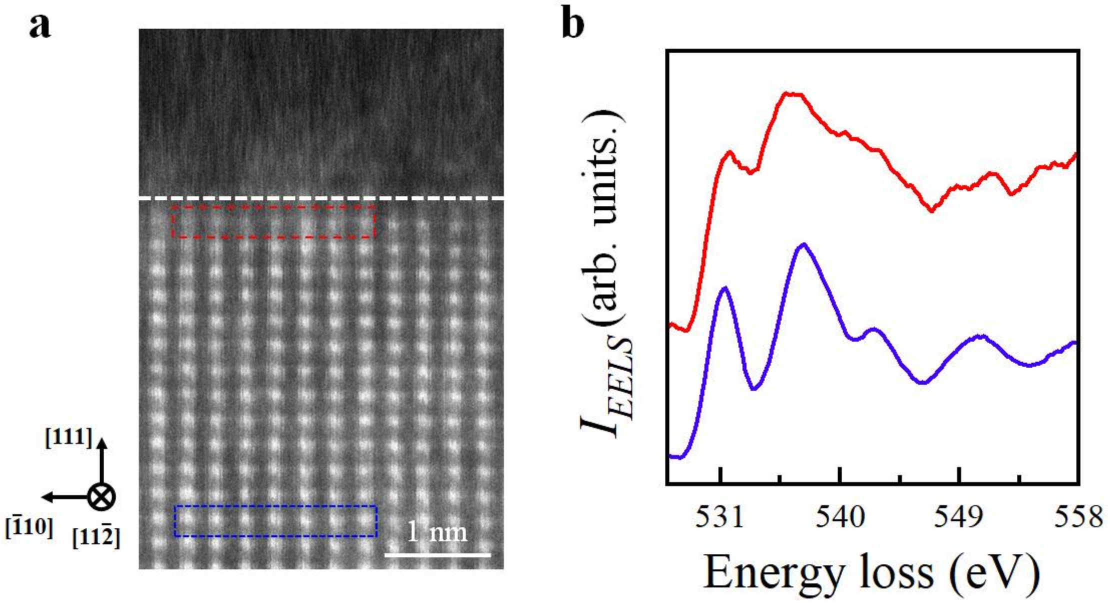

Figure S3 │ EELS spectra of oxygen K edge. a, The HAADF-STEM image of LAO/KTO(111) sample. **b**, The EELS spectrums for area close to (red curve corresponding to the red box in panel **a**) and far away (blue curve, blue box in panel **a**)

from the interface. The oxygen K edge absorption is much weaker for the red curve, implying a high density of oxygen vacancies at the interface.

To characterize the oxygen vacancies, the electron energy loss spectroscopy (EELS) spectra of oxygen K edge was taken for area close to and far away from the LAO/KTO interface. The weaker oxygen K edge absorption implies a higher density of oxygen vacancies at the interface.

According to STEM measurements, the lattice structure of interfacial KTO layers with ferroelectric polarization is sketched in Fig. S4. K atoms are displaced relative to the Ta-O lattice such that the centers of positive and negative ions are displaced relative to each other—the defining characteristic of ferroelectricity.

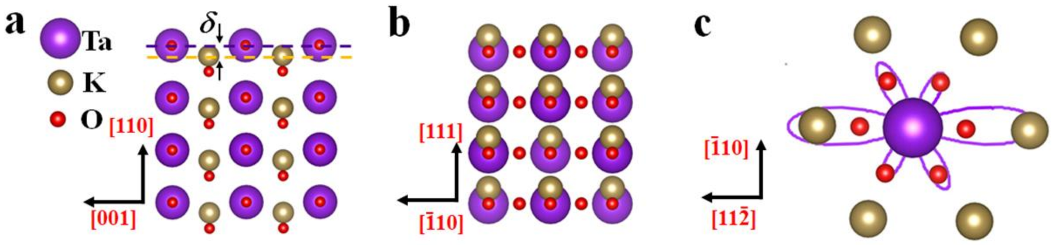

Figure S4 | Projections of polar KTO lattice in the (1-10) (a), (11-2) (b), and (111) (c) planes according to STEM images. The purple solid line represents the angle-dependent second harmonic generation (SHG) intensity $I_x^{2\omega}(\phi)$ at room temperature.

2. SHG signals of KTO substrate

Since the inversion symmetry of KTO(111) is broken at its surface, the bare KTO(111) substrate, in principle, could allow SHG to emerge. However, by comparing the SHG signals from the LAO/KTO(111) sample and the KTO(111) substrate (Fig. S4), it is confirmed that the KTO(111) substrate itself does not generate measurable SHG signal, and the measured SHG signal of LAO/KTO(111) therefore originates from the heterointerface.

To examine whether the annealing process of KTO substrate introduces oxygen vacancies and electric polarization, we carried out the following experiment. The KTO(111) substrate was heated to 300°C under conditions identical to those used for LAO film deposition. The annealed KTO(111) substrate produced only a weak SHG signal (Fig. S5). We also attempted to write patterns with tip voltage ramped all the way up to ±50 V but observed no contrast in either piezoresponse force microscopy (PFM) or electrostatic force microscopy (EFM) images after writing, indicating that the electric polarization was too weak to be detected by PFM or EFM.

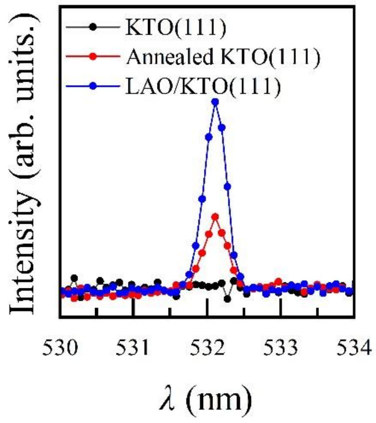

Figure S5 | SHG signals for the KTO(111) substrate, annealed KTO(111) substrate, and LAO/KTO(111) sample. The wavelength of the laser is 1064 nm and the SHG light shall have a wavelength of 532 nm.

As proposed in the main text, oxygen vacancies at the LAO/KTO interface presumably affect the relative rotation of the oxygen octahedra, as well as the Ta-O-Ta bond length and angle, thereby inducing interfacial ferroelectricity. For the annealed KTO(111) substrate, the same mechanism may also operate: oxygen vacancies generated at the surface during annealing give rise to a weak electric polarization. This is consistent with literature reports that oxygen vacancies can enhance electric polarization or piezoelectricity in perovskite oxide films[45, 46].

3. SHG signals during air annealing

We annealed the LAO/KTO(111) samples in air to examine the influence of oxygen vacancies. While ramping the temperature from room temperature to 450°C, I_{SHG} manifests a pronounced peak around 300°C for both LAO/KTO(111) samples (Fig. S6), indicative of a change in the structure or chemical stoichiometry. This behavior complicates the data analysis and warrants further investigation. Nevertheless, the SHG signal survives the annealing process, albeit with reduced intensity, and the SHG symmetry remains unchanged upon returning to room temperature.

It is not surprising that the ferroelectric order varies with the concentration of oxygen vacancies. As noted in the original manuscript, ferroelectricity at the LAO/KTO interface must be correlated with oxygen vacancies at the interface (Fig. 1). Thus, it is consistent with expectations that the strength of electric polarization changes with oxygen vacancy density. However, the key point here is that the observed SHG signal originates from ferroelectric order, rather than directly from oxygen vacancies.

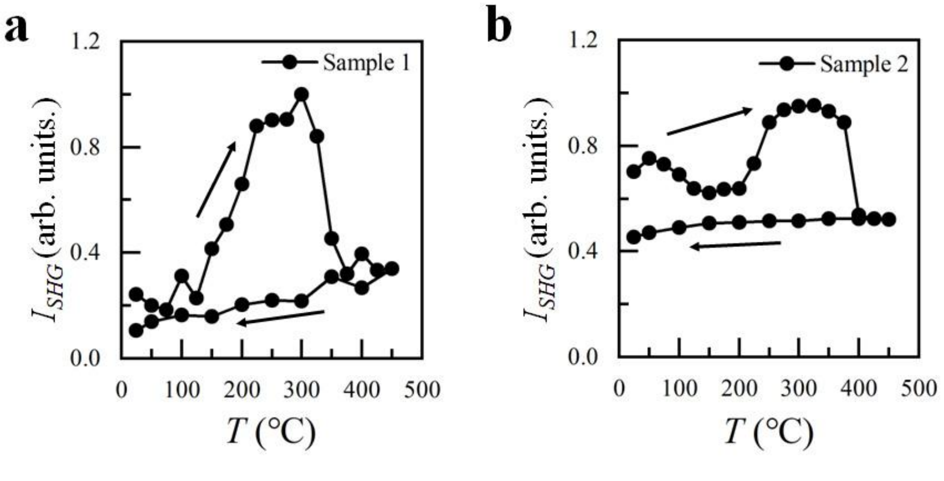

Figure S6 | SHG intensity of LAO/KTO(111) during air annealing for the sample #1 (a) and #2 (b). I_{SHG} manifests a pronounced peak during heating, indicative of a change in the structure or chemical stoichiometry.

In our SHG optical configuration, both the incident laser and the generated second-harmonic light are normal to the film surface, and the polarizations of both are in-plane (Fig. 2a). As a result, our SHG setup is sensitive only to the in-plane component of electric polarization. The charge transfer between LAO and KTO, and the resultant electric polarization, are orientated along the out-of-plane direction. Therefore, the SHG signal in our measurements is mainly contributed by the in-plane component of ferroelectric polarization along the [110] direction (Fig. 1). Furthermore, we demonstrated in Fig. S9 that oxygen vacancies in LAO/MgO(001) generate no detectable SHG signal. Therefore, the SHG signal of LAO/KTO(111) shall be attributed to ferroelectric order.

4. Time decay of PFM contrast after pattern writing

Another example of ferroelectric pattern is shown in Fig. S7, which was created by applying ±10 V on the PFM tip. The area of both the inner and outer square is smaller than the pattern shown in Fig. 3, demonstrating that the flipping of ferroelectric polarization can be routinely done.

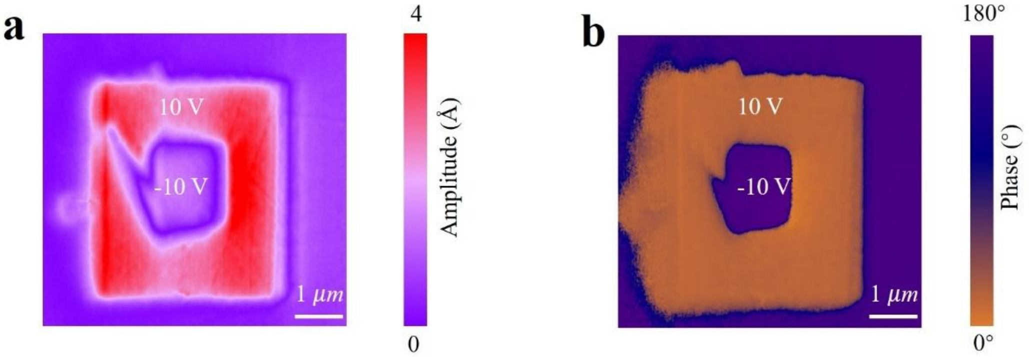

Figure S7 │ Another example of ferroelectric pattern written by PFM. The inner and outer squares are 2 × 2 and 4 × 4 μm^2 written by applying +10 V and -10 V to the PFM tip respectively.

To distinguish possible mechanisms underlying the PFM contrast, such as ferroelectric polarization or redistribution of oxygen vacancies, the stability of the PFM pattern was investigated (Fig. S8). After writing concentric square patterns with +15 and -15 V, the PFM amplitude decreases rapidly from 2.7 to 1.5 in the first 1 hour, then more gradually from 1.5 to 0.5 between 1 and 12 hours, after which it stabilized. PFM images taken at 10, 720, and 1440 minutes (upper inset) corroborate these trends. In stark contrast, EFM images acquired at the same time intervals (lower inset) show rapid contrast decay, vanishing by 720 minutes (note the identical coloring of the central square and its surroundings at 720 and 1440 minutes). From these observations, we infer that the persistent PFM signal originates from ferroelectric polarization, reversible via tip gating; the decaying components of the PFM and EFM signals likely relate to oxygen-vacancy redistribution.

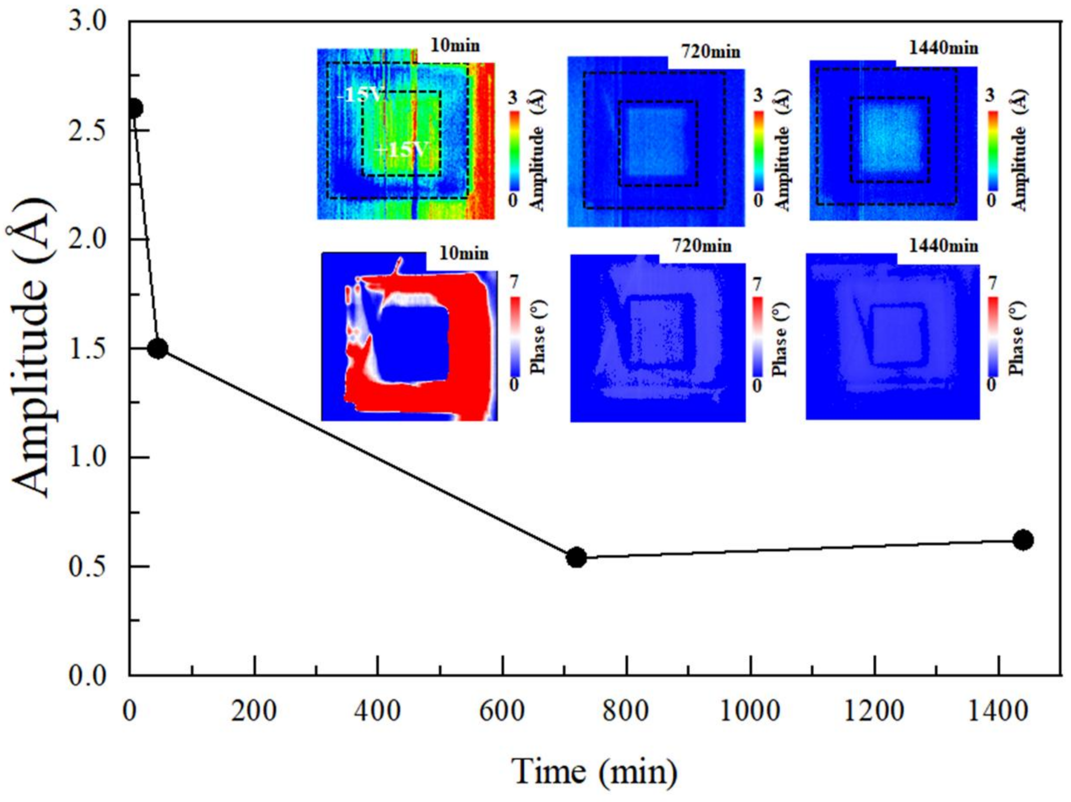

Figure S8 | Time decay of the PFM amplitude. A concentric square pattern was written onto LAO/KTO(111) sample at time zero, and the PFM amplitude at the center square was monitored over time. A substantial PFM signal persists beyond 24 hours, indicating a stable ferroelectric polarization. The corresponding PFM and EFM images

after 10, 720, and 1440 minutes are shown in the upper and lower inset panels, respectively. While the PFM contrast remains with time, the EFM contrast diminishes by 720 minutes, supporting the interpretation that the EFM signal arises from surface charge or oxygen vacancy effects, whereas the PFM signal originates from ferroelectricity.

5. Control experiment on $LaAlO_3$/MgO(001) (LAO/MgO)

We performed a comparative experiment on LAO/MgO(001) to verify that the LAO film itself, or its interface with another oxide, would not trivially produce the PFM and SHG signals observed in LAO/KTO(111).

The LAO/MgO(001) film was grown under identical conditions to the superconducting LAO/KTO(111) samples. Applying ±30 V on the PFM tip—well above the voltage applied for LAO/KTO(111)—we wrote a concentric square pattern on LAO/MgO.

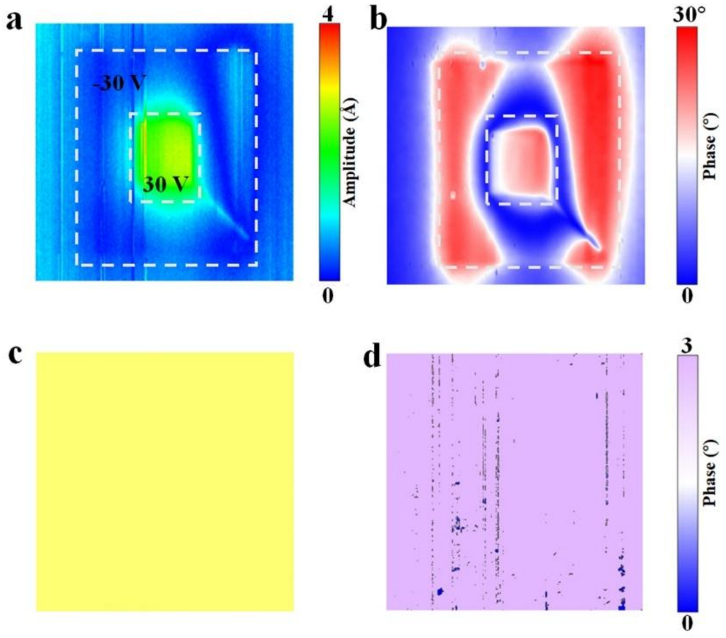

Figure S9 | PFM (a), EFM (b), and SHG (c) images of the LAO/MgO(001) sample after writing a concentric square pattern with the PFM tip. No SHG signal was

detected, and the EFM contrast decayed within 48 hours (d), in sharp contrast to that observed on LAO/KTO(111).

By comparing the results for LAO/KTO(111) (Figs. 3 and S8) and LAO/MgO(001) (Fig. S9), PFM and EFM contrast appear in LAO/MgO but vanish within 48 hours, indicating that these signals likely arise from surface charging or oxygen-vacancy effects. The EFM signal of LAO/KTO(111) also decays over time, presumably for the same reason. However, the time-stable component of the LAO/KTO PFM signal points to the presence of switchable ferroelectric polarization. No SHG signal was detected from LAO/MgO, even immediately after pattern writing, in sharp contrast to the stable SHG signal from LAO/KTO. This indicates that surface charging or oxygen vacancies do NOT generate SHG signals, and that the SHG signal from LAO/KTO originates from ferroelectric polarization.

6. Hysteresis loops of another two LAO/KTO(111) samples

To demonstrate that the tip gating is universally applicable to LAO/KTO(111) samples, we carried out the gating of another two samples using PFM tip in a way identical to that is shown in Fig. 4.

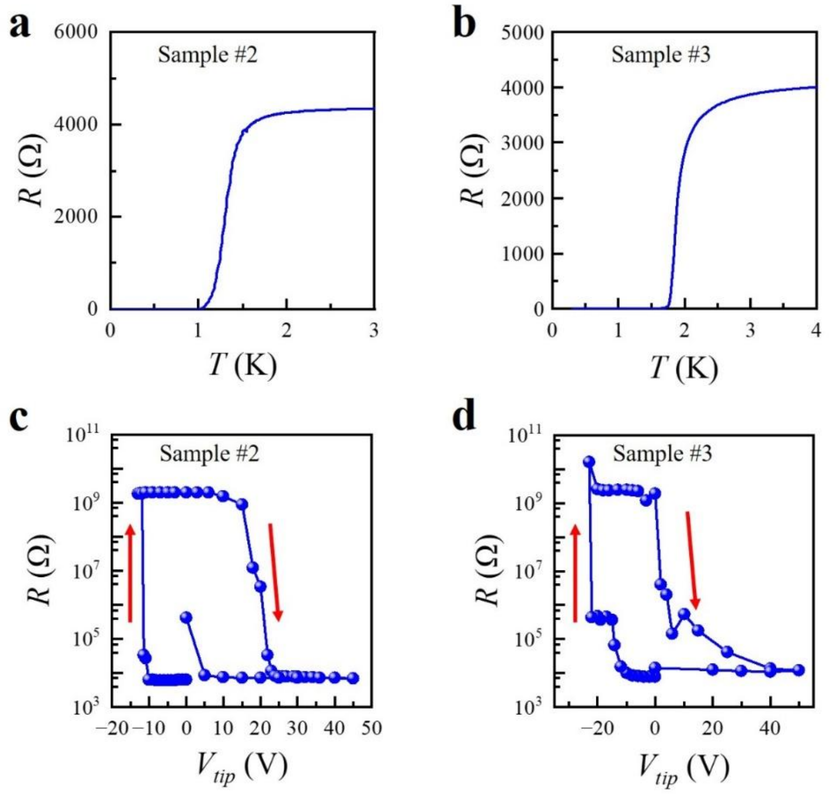

Figure S10 │ The hysteresis loops of conductivity for another two LAO/KTO(111) samples. **a,** and **b**, $R(T)$ manifest clear superconducting transitions but with different T_c for sample #2 and #3. **c**, and **d**, Despite of differences in T_c, both samples show hysteresis behavior as the measured area are modulated by the PFM tip. These results are qualitatively the same to those in Fig. 4, showing that the strong coupling between ferroelectricity and conductivity is universal for LAO/KTO(111) samples.

The resistance of two LAO/KTO(111) samples behaves hysterically in response to the tip gating voltage V_{tip}. Despite the difference in coercivity, both samples switch between a high and low resistance state as V_{tip} ramps, identical to the sample shown in Fig. 4. The hysteresis loop of the interfacial conductivity is a manifestation of ferroelectric hysteresis loop.

7. Hall effect on LAO/KTO(111)

We have measured the Hall effect of LAO/KTO(111) (Fig. S11) in an attempt to reveal the underlying cause for the giant resistance change and modulation of

superconductivity. Since ramping the magnetic field takes hours, the Hall effect measurements were performed 10 hours after the writing when a stable state had been reached. During this time, the effect decays and the change for the high/low resistance state is reduced to roughly 355 times. The carrier mobility, μ_{Hall}, retrieved from the measured longitudinal resistance and Hall coefficient, is 261 times smaller in the non-superconducting high resistance state than in the superconducting low resistance state (Fig. S11d). In contrast, the carrier density, n_{2D}, changes by a factor of 1.36 in the meantime (Fig. S11c). Thus, the reduction in Hall mobility is seemingly the prime reason for the loss of superconductivity. However, it should be noted that the mean free path for the highest resistance state (V_{tip} = -21 V) is shorter than the lattice constant (Fig. 4), indicating that percolative conductivity plays a major role. Thus, for the high resistance state, the retrieved Hall number is under influence of percolative connectivity and should be interpreted with caution.

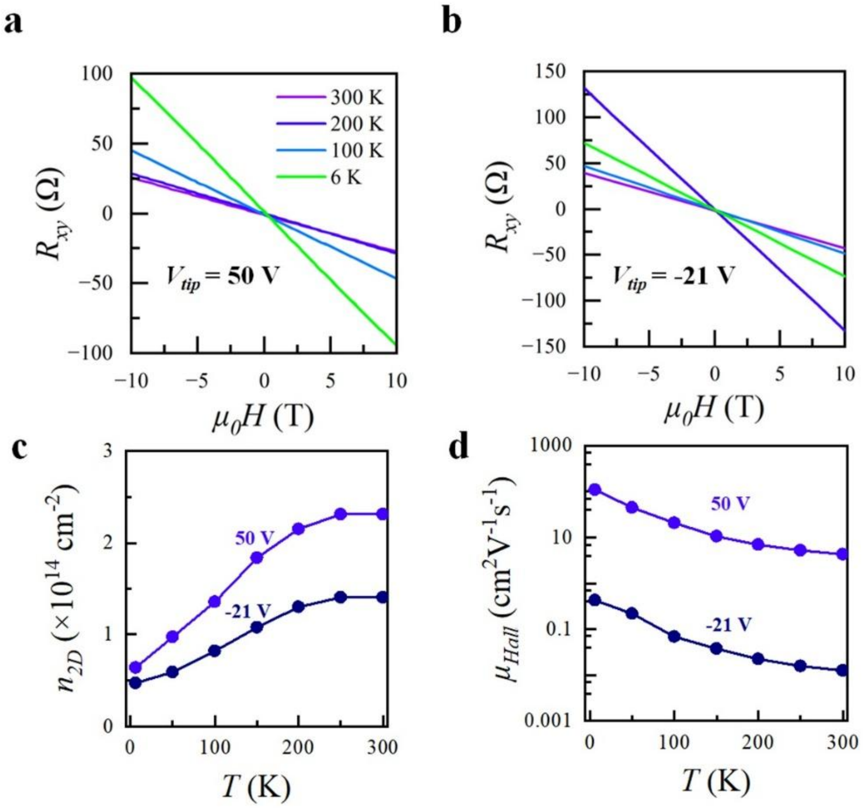

Figure S11 │ The Hall effect of the LAO/KTO(111) sample. **a,** and **b**, Two representative Hall effect measurements for the LAO/KTO(111) sample after gated

under 50 and -21 V, respectively. **c**, The sheet electron density, n_{2D}, as a function of temperature. **d**, The corresponding electron mobility $\mu_{Hall}(T)$.

8. Ferroelectricity in the superconducting state

To unambiguously demonstrate the coexistence of superconductivity and ferroelectricity, we devoted substantial effort to lowering the base temperature of our SHG measurement setup.

To reduce the heating from the laser beam, we replaced the picosecond laser with a femtosecond laser and lowered the laser power to only 1.5 mW for the measurements shown in Fig. S12, compared with 83 mW in Figs. 2e and 2f. Despite this drastically reduced laser intensity, the SHG signal in Fig. S12 remains significant. With these improvements, the base temperature of the LAO/KTO(111) film reaches 1.62 K—well below its superconducting temperature. During the SHG measurements at 1.62 K, we simultaneously monitored the resistance to ensure that the film stayed in the zero-resistance state, thus ruling out any light-induced transition out of superconductivity.

As shown in Fig. S12a, the resistance of the LAO/KTO(111) sample drops to zero at approximately T = 1.75 K (the $R(T)$ curve was measured both in a dilution refrigerator and in the cryostat equipped with SHG optics). Concurrently, an SHG signal is clearly detected at T = 1.62 K, when the sample is in the superconducting state. Here the wavelength of the incident laser is 1031$\pm$7 nm and a pronounced peak appears near 517 nm, corresponding to the SHG signal. The SHG intensity, $I_{SHG}(T)$, remains roughly the same at elevated temperatures (Figs. S12b and S12c), consistent with Fig. 2d. These results confirm that ferroelectric polarization is robust from room temperature all the way down to the superconducting regime.

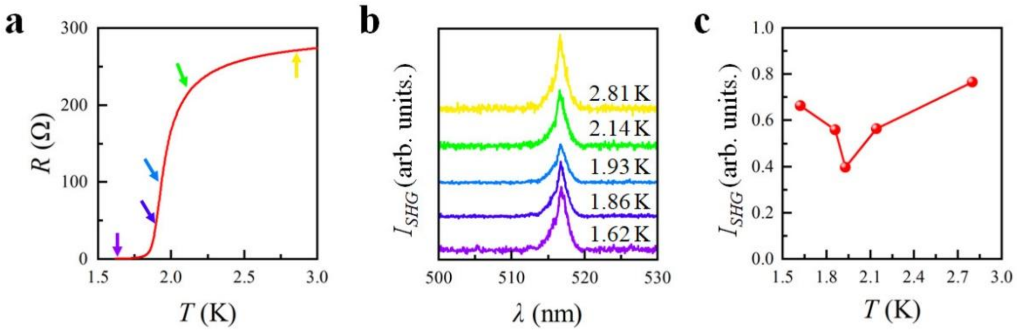

Figure S12 | Low-temperature SHG experiments confirm the coexistence of ferroelectricity and superconductivity in LAO/KTO(111). **a**, Temperature dependence of the resistance $R(T)$, showing the onset of zero-resistance at ~1.75 K. The arrows indicate the temperatures at which SHG measurements were performed. **b**, With an incident laser wavelength of 1031±7 nm, a pronounced peak appears near 517 nm, corresponding to the SHG signal. SHG spectra acquired from T = 1.62 to 2.81 K provide clear evidence of ferroelectric order persisting within the superconducting state. **c**, Temperature dependence of the SHG intensity $I_{SHG}(T)$, demonstrating that ferroelectricity remains robust down to 1.62 K and thus coexists with superconductivity.

9. Absence of charge trapping/detrapping

Switching experiments provide evidence against charge trapping as the dominant mechanism (Fig. S13). The critical distinction lies in the hysteretic behavior: charge trapping or detrapping would produce volatile, non-hysteretic resistance changes that track the applied voltage instantaneously. In contrast, our data reveals a completely different, strongly hysteretic behavior:

1. Stable bistates above the coercive voltage. Once the voltage applied to the PFM tip during scanning exceeds the coercive voltage, the LAO/KTO(111) film switches into one of two stable states—low or high resistance—depending solely on the voltage polarity (Fig. S13a).
2. Non-volatile switching to the high-resistance state. Starting from the low-resistance state at 0 V, stepwise ramping V_{tip} down to -50 V drives the film to

the high-resistance state. When V_{tip} is subsequently ramped back to 0 V, the resistance remains locked, demonstrating clear non-volatile memory (Fig. S13c).

3. Non-volatile switching to the low-resistance state. Starting from either a high (Fig. S13d) or low (Fig. S13f) resistance state at 0 V, increasing V_{tip} stepwise to +50 V forces the film into the low-resistance state, which remains stable when V_{tip} is returned to 0 V.
4. Retention of intermediate states. When V_{tip} is swept to a value corresponding to an intermediate resistance state (Figs. S13b and S13e), reducing V_{tip} back to 0 V preserves this intermediate state as well.

This pronounced hysteresis, robust retention, and non-volatile memory observed here are hallmark signatures of ferroelectric polarization switching. Charge trapping, by contrast, would cause the resistance to relax upon field removal—behavior absent in Fig. S13. These results therefore provide direct experimental evidence that ferroelectric switching, rather than charge trapping/detrapping, governs the resistance modulation.

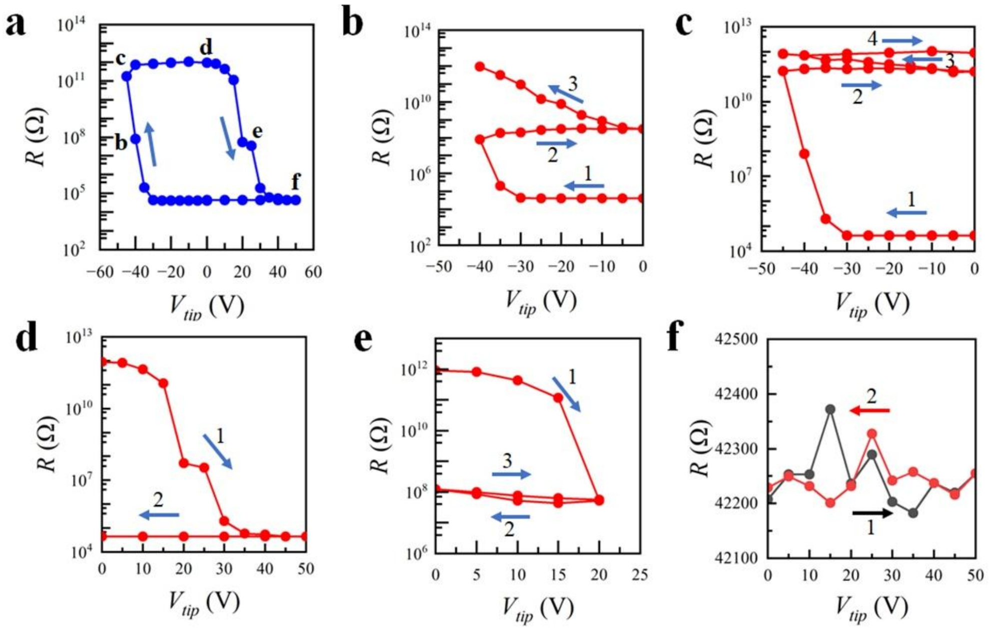

Figure S13 | Absence of charge trapping/detrapping in LAO/KTO(111). a, The longitudinal resistance manifests clear hysteresis behavior in response to PFM tip writings at 300 K. **b-f**, The tip voltage is ramped from 0 V step-by-step to a designated value V_{max} and then ramped back to 0 V. The sequence of voltage ramping is indicated

by the arrows and numberings. The value of V_{max} for panels **b** to **f** are indicated by the labels from “b” to “f” in panel **a**, respectively. Apparently, the hysterical behavior of $R(V_{tip})$ is correlated with ferroelectric hysteresis, rather than charge trapping/detrapping.
